# The Relationship between Body Mass Index and Frontal QRS-T Angle in Pregnant Women Undergoing Cesarean Section with Spinal Anesthesia

**DOI:** 10.3390/medicina60081277

**Published:** 2024-08-07

**Authors:** Mehmet Tercan, Tugba Bingol Tanriverdi, Nurseda Komurcu, Alev Esercan, Ahmet Kaya, Erhan Ozyurt, Zulkif Tanriverdi

**Affiliations:** 1 Department of Anesthesiology and Reanimation, University of Health Science Mehmet Akif Inan Research and Training Hospital, Sanliurfa 63040, Turkey; tuggbabingol@gmail.com (T.B.T.); ahmetkayamd@yahoo.com (A.K.); 2 Department of Anesthesiology and Reanimation, Sanliurfa Research and Training Hospital, Sanliurfa 63200, Turkey; nursedakk@outlook.com; 3 Department of Obstetrics and Gynecology, Sanliurfa Research and Training Hospital, Sanliurfa 63200, Turkey; alevesercan@gmail.com; 4 Department of Anesthesiology and Reanimation, University of Health Science Antalya Research and Training Hospital, Antalya 07100, Turkey; eozyurt@hotmail.com; 5 Department of Cardiology, Faculty of Medicine, Harran University, Sanliurfa 63050, Turkey; ztverdi@gmail.com

**Keywords:** anesthesia, pregnancy, cesarean section, myocardial repolarization, frontal QRS-T angle

## Abstract

*Background and objectives*: The frontal QRS-T angle is a novel parameter of myocardial repolarization. Weight gain during pregnancy and physiological changes during a cesarian section may affect the frontal QRS-T angle. We aimed to assess the effect of body mass index (BMI) on the frontal QRS-T angle in pregnant women undergoing cesarean section with spinal anesthesia. *Method and materials*: This study included 90 pregnant women. BMI was calculated for all pregnant women. The study population was divided into two groups: BMI < 30 (n = 66) and BMI ≥ 30 (n = 24). QT interval measurements and the frontal QRS-T angle were obtained from the report of an electrocardiography machine. *Results*: It was found that the pre-operative and post-operative frontal QRS-T angle (*p* = 0.045 and *p* = 0.007) and QTc interval (*p* = 0.037 and *p* < 0.001) were higher in pregnant women with a BMI ≥ 30 than in pregnant women with a BMI < 30. In addition, when compared to pre-operative values, the post-operative frontal QRS-T angle (from 24.0 [20.0–41.5] to 34.5 [19.5–50.0], *p* = 0.031) and QTc interval (from 420.6 ± 13.3 to 431.7 ± 18.3, *p* = 0.010) were increased in the BMI ≥ 30 group, whereas no significant post-operative increase was observed in the BMI < 30 group. In correlation analysis, BMI was positively correlated with the frontal QRS-T angle and QTc interval. *Conclusions*: The frontal QRS-T angle and QTc interval were importantly increased in pregnant women with a BMI ≥ 30 than in pregnant women with a BMI < 30. Also, after cesarean section operation with spinal anesthesia, the frontal QRS-T angle and QTc were increased significantly in the BMI ≥ 30 group, whereas no significant change was observed in the BMI < 30 group. Therefore, it is suggested to perform close post-operative monitoring in pregnant women with a BMI ≥ 30 undergoing cesarean section with spinal anesthesia.

## 1. Introduction

Cardiovascular diseases are a major cause of non-obstetric maternal mortality and morbidity during pregnancy and the postpartum period, and have gradually increased over time. The increased risk of morbidity and mortality has been attributed to older pregnancy, pre-existing comorbidities such as hypertension, diabetes mellitus, and obesity, and the growing survival rate of pregnant people with congenital heart disease [1].

Many positional, physiological, and anatomical changes occur during pregnancy to meet the increased metabolic demand of pregnant organs and the fetus, especially in the cardiovascular system. Heart rate, plasma volume, cardiac output, and sympathetic tonus are increased, whereas vascular resistance and blood pressure are reduced. Also, the heart changes its position and shifts to the left and upward due to the elevation of the diaphragm by the gravid uterus [2]. All these changes may be seen more clearly in pregnant women with more weight gain and a higher body mass index (BMI) during pregnancy, and result in cardiac electrical remodeling and impair the myocardial depolarization/repolarization. Consequently, susceptibility to arrhythmias may be increased during pregnancy.

In addition to physiological and anatomical changes in pregnancy, the anesthesia technique may also have an impact on myocardial repolarization [3]. Spinal anesthesia is the preferred anesthetic technique for elective cesarian deliveries due to its advantages compared to general anesthesia [4]. Nevertheless, spinal anesthesia may result in different complications, such as reduced sympathetic activity, systemic vascular resistance, and impairment in myocardial repolarization [2,5].

Myocardial repolarization is traditionally assessed by QT interval measurements in surface electrocardiography (ECG) [6,7]. A prolonged QT interval has been found to be an important predictor of malign arrhythmias and sudden cardiac death [8]. Recently, the frontal QRS-T angle has arisen as a novel parameter of myocardial repolarization and depolarization heterogeneity and gained more interest over the QT interval [9]. It is the angle difference between myocardial depolarization and repolarization and is measured as the subtraction of the T axis from the QRS axis. Because these two axes are already available in ECG reports, the frontal QRS-T angle can quickly and easily be measured from an ECG [10]. Studies demonstrated that the frontal QRS-T angle had a significant prognostic role in predicting high cardiac-risk patients [9,10].

It was reported that the QT interval was prolonged during pregnancy [11]. It was also detected that spinal anesthesia increased QT interval measurements in pregnant women within ≥42 weeks of undergoing cesarean operation [2]. However, no study has investigated the change in the frontal QRS-T angle in pregnant women undergoing cesarean operation in the literature. This study aimed to evaluate the effect of BMI on the frontal QRS-T angle in pregnant women undergoing cesarean section with spinal anesthesia.

## 2. Methodology

### 2.1. Patient Selection

The study design was approved by the Harran University Clinical Research Ethics Committee (date: 17 October 2022, number: HRÜ/22.20.27), and informed consent was taken from all pregnant participants. This study started on 20 October 2022 at the obstetrics clinic of the training and research hospital and was completed on 15 February 2023.

Pregnant women undergoing an elective cesarian delivery were enrolled in the study. The inclusion criteria were pregnant women ≥18 years old with an American Society of Anesthesiologists (ASAs) score of II who were scheduled for an elective cesarean section. Known previous cardiac disease, hypertension, diabetes mellitus, drug use affecting the QT interval, electrolyte disorders, severe anemia, and a baseline complete or incomplete bundle brunch block were exclusion criteria. After informing the patients, 90 patients who met the study criteria and agreed to participate in the study were included. Information about the patients was obtained from the medical histories of the patients, archive file records, and the hospital information registration system. Baseline clinical and laboratory characteristics, weight, height, duration of the operation, gestational week, and previous number of cesarian deliveries were recorded for all patients. After this information was obtained, the BMI of all cases was calculated as the ratio between the weight and square height [12]. According to the BMI, the subjects were assigned into two groups in the current study: group I, BMI < 30 (n = 66), and group II, BMI ≥ 30 (n = 24). All pregnant women received the same anesthesia protocol and followed up for hemodynamic and electrocardiographic variables.

### 2.2. Anesthesia Management

Because it was shown that spinal anesthesia is a better option for elective cesarean section owing to its benefits for maternal and fetal outcomes, spinal anesthesia was used in all cases of the current study [4]. Vascular access was achieved; then, pregnant women were taken into the operating room and monitored for heart rate and mean arterial pressure. Pre-operative hydration with Ringer Lactate solution at 10 mL/kg was initiated. Following this, the pregnant woman was placed into the sitting position, and a spinal block was conducted by administering 12.5 mg of 0.5% bupivacaine (Marcain^®^ spinal heavy, 0.5%, 4 mL ampule, AstraZeneca) with a 26 G spinal needle through the L3-4 intervertebral space in the sitting position. After the anesthesia induction, the patients were placed in the supine position with support on the back and hips to achieve a 15-degree left lateral position. The sensory block was evaluated with the pinprick test, and the operation started when the sensory block at the T4-6 level was achieved. Heart rate and mean arterial pressures were monitored during the procedure. In the pre-operative period and the 1st, 5th, 10th, and 20th min during the operation, post-operative heart rate and mean arterial pressures were recorded.

### 2.3. Electrocardiographic Evaluation

**Figure 1:**
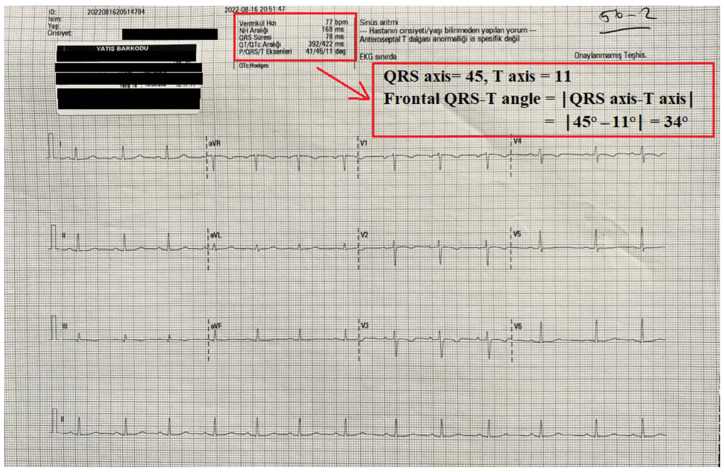
An illustration of the automatic record of an ECG device and the calculation of the frontal QRS-T angle from this record.

Twelve-lead electrocardiograms (ECGs) (Nihon Kohden, Tokyo, Japan) were obtained from all patients before and after surgery. The automated reports of all ECGs were also recorded. QT interval measurements and the frontal QRS-T angle were taken from the report of the ECG machine. The QT and QTc interval were measured as previously defined [7,13]. The frontal QRS-T angle was calculated as the absolute difference between the QRS axis and T axis (frontal QRS-T angle =│QRS axis − T axis│). If the calculated angle exceeded 180°, it was subtracted from 360° [9]. An illustration of the report of an ECG device and the calculation of frontal QRS-T angle is shown in Figure 1.

### 2.4. Statistical Analysis

Statistical analysis was applied with the SPSS 23.0 program. The normality test was analyzed with the Kolmogorov–Smirnov test, histograms, skewness, kurtosis, and Q–Q plots. Continuous data with normal distribution are presented as the mean ± standard deviation, while continuous data without normal distribution are presented as the median (25–75th interquartile range). The comparison of two independent groups was performed with Student’s *t* test or the Mann–Whitney U test according to the distribution. A comparison of pre-operative and post-operative data (at two different time points) within the same group was performed with the paired sample t-test or Wilcoxon test. Repeated measures analysis of variance (ANOVA) was used for repeated measurements more than two times. Pearson or Spearman correlation coefficients were used for the correlation analysis. A *p* value of <0.05 was accepted as significant.

## 3. Result

The mean age of the pregnant women was 27.2 ± 4.2 years, while the mean BMI was 28.3 ± 2.7 kg/m^2^. Sixty-six of the pregnant women had a BMI of <30, whereas 24 had a BMI of ≥30.

The comparison of the basal and clinical characteristics according to BMI is shown in Table 1. Age (*p* = 0.802), gravidity (*p* = 0.156), duration of surgery (*p* = 0.903), gestational week (*p* = 0.473), and number of previous cesarean sections (n = 0.672) were similar between the two groups.

The blood pressure and heart rate measurements at each time point during surgery are listed in Table 2. No important difference was observed between the groups regarding blood pressure and heart rate measurements when comparing the values at the same time points. In both groups, I and II, heart rate increased at least once and then gradually decreased in the repeated measurements. Mean arterial pressure also decreased significantly compared to pre-operative values.

QT interval and frontal QRS-T angle measurements are shown in Table 3. The pre-operative and post-operative QT intervals were similar between the two groups. However, it was found that pre- and post-operative QTc (*p* = 0.037 and *p* < 0.001) and the frontal QRS-T angle (*p* = 0.045 and *p* = 0.007) were significantly higher in pregnant women with a BMI ≥ 30 than in pregnant women with a BMI < 30. On the other hand, compared to pre-operative values, post-operative QT (from 355.6 ± 22.1 to 371.8 ± 32.9, *p* = 0.008), QTc (from 420.6 ± 13.3 to 431.7 ± 18.3, *p* = 0. 010), and the frontal QRS-T angle (from 24.0 [20.0–41.5] to 34.5 [19.5–50.0], *p* = 0.031) were significantly increased in the BMI ≥ 30 group, whereas no significant post-operative increase was detected in the BMI < 30 group.

**Figure 2:**
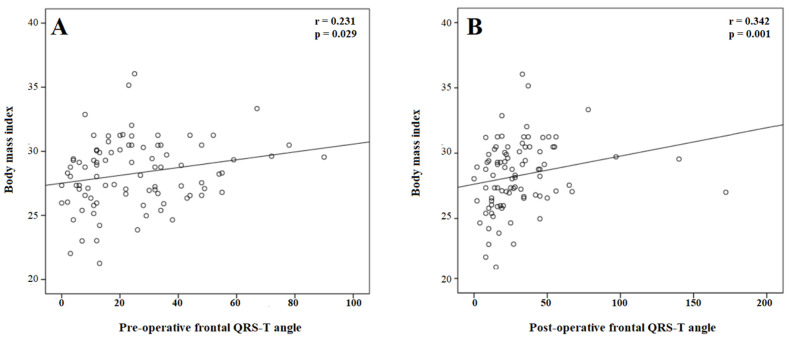
The correlation of body mass index with the pre- (**A**) and post-procedural (**B**) frontal QRS-T angle.

For the correlation analysis, BMI was positively correlated with the pre-operative (r = 0.220, *p* = 0.037) and post-operative (r = 0.248, *p* = 0.018) QTc interval. In addition, BMI was positively correlated with the pre-operative (r = 0.231, *p* = 0.029) and post-operative (r = 0.342, *p* = 0.001) frontal QRS-T angle (Figure 2).

## 4. Discussion

In the current study, the effect of pregnancy and spinal anesthesia on the frontal QRS-T angle according to the BMI in pregnant women who underwent a cesarean section was investigated. Our main findings were as follows: (I) the pre-procedural and post-procedural QTc interval and frontal QRS-T angle were significantly higher in pregnant women with a BMI ≥ 30 than in pregnant women with a BMI < 30, (II) spinal anesthesia significantly increased the QT, QTc and frontal QRS-T angle in the BMI ≥ 30 groups, whereas it had no effect on these parameters in the BMI < 30 group, and (III) BMI was significantly correlated with pre-procedural and post-procedural QTc and frontal the QRS-T angle. This is the first report to investigate the link between spinal anesthesia, BMI, and frontal QRS-T angle in pregnant women undergoing a cesarean section.

The heart undergoes many changes during pregnancy due to the increased metabolic demands of the mother and the developing fetus. In addition to hemodynamic effects and functional changes, pregnancy leads to significant structural changes in the heart, including ventricular enlargement and an increase in myocardial wall mass [14]. In addition, the heart changes position, shifting to the left and upward due to increased weight gain and the elevation of the diaphragm by the gravid uterus [2]. These changes are expected to become more pronounced as weight gain increases. After childbirth, these changes return to pre-pregnancy levels within about 6 weeks. However, anatomical and physiological changes during labor and the type of anesthesia used itself may also affect the cardiovascular system. Therefore, a detailed assessment of all pregnant women for cardiovascular disease and risk is important to prevent maternal and fetal complications.

Previous studies have shown that pregnancy increases the measurement of the QT interval [11,15]. It has also been shown that this increase in the QT interval measurement occurs in late pregnancy (the third trimester) [16]. The main causes of QT prolongation in pregnancy are changes in ventricular repolarization due to hormonal status and ventricular enlargement as a result of increased volume overload [17]. In addition, an increased BMI shifts the position of the heart to the left, which also alters ventricular repolarization. Therefore, an increase in ventricular repolarization parameters in the last trimester, when weight gain is greatest, is an expected situation. Although previous studies have demonstrated the effect of pregnancy on the QT interval, no study has investigated the effect of BMI on ventricular repolarization parameters in pregnant women. It has been shown, for the first time, that the QTc interval was significantly longer in pregnant women with a BMI ≥ 30 than in those with a BMI < 30. Our results suggest that repolarization alterations are more common in pregnant women with higher weight gain. Supporting the findings of the current study, a previous study reported that pregnant women living at moderate altitudes had significantly longer QT parameters than those living at sea level [18]. These findings suggest that the QT interval prolongation in pregnancy may also be due to some other environmental factors, and this issue should be further investigated.

In the current study, the frontal QRS-T angle, which is a novel marker of ventricular repolarization heterogeneity, was also evaluated. The frontal QRS-T angle has been shown to be more reproducible and less susceptible to noise than the QT interval and, therefore, superior to the QT interval [19,20]. In the current study, it was observed that frontal QRS-T angle was also importantly higher in pregnant women with a BMI ≥ 30 than in pregnant women with a BMI < 30. Furthermore, BMI was positively correlated with the pre- and post-operative frontal QRS-T angle. It can be concluded that the alteration in ventricular repolarization is expected to be greater in pregnancies with higher BMI.

Spinal anesthesia is the preferred anesthetic technique for an elective cesarean section because of its advantages over general anesthesia [4]. However, physiological changes during labor and the type of anesthesia itself may also affect the cardiovascular system and alter ventricular repolarization [3]. Previous studies have shown that QT interval measures are increased in pregnant women undergoing a cesarean section with spinal anesthesia [2,21]. The possible mechanisms are pain, anxiety, and bleeding during labor and the hemodynamic effect of spinal anesthesia [21]. In the current study, the effect of spinal anesthesia according to BMI classification was assessed. It was found that compared with pre-operative values, post-operative QTc and the frontal QRS-T angle were importantly increased in the BMI ≥ 30 groups, whereas no significant post-operative increase was detected in the BMI < 30 group. Our results suggest that spinal anesthesia leads to a greater alteration in myocardial repolarization in pregnant women with a BMI ≥ 30. Considering that QTc and the frontal QRS-T angle are the parameters that predict arrhythmia, it can be concluded that pregnant women with a BMI ≥ 30 undergoing a cesarean section under spinal anesthesia should be closely monitored for arrhythmia during the operative and early post-operative periods.

Although bupivacaine is the most commonly used anesthetic agent for cesarean section under spinal anesthesia, it has been reported that bupivacaine may have cardiotoxic effects, especially at higher doses [22,23]. The cardiotoxic effect of bupivacaine has been attributed to its high affinity for myocardial Na+ channels [24]. In a previous study, Hanbeyoglu et al. compared the effect of different doses of bupivacaine (10 mg vs. 15 mg) on the QTc interval [25]. They found that QTc interval prolongation was dose-dependent and significantly higher with 15 mg of bupivacaine than with 10 mg. In addition, Deniz et al. showed that 10 mg of bupivacaine did not affect QT dispersion in cesarean sections under spinal anesthesia [26]. These results demonstrate that low doses of bupivacaine have a less significant effect on the QT interval. In the present study, bupivacaine was used at a dose of 12.5 mg in all patients. Therefore, it is suggested that the effect of bupivacaine is similar in both BMI groups in the current study, and increased BMI is the main cause of the difference between the two groups.

The present study had some limitations. First, the number of patients was small. Second, performing a serial ECG recording and determining when the QTc and frontal QRS-T angle returned to normal after surgery could have made an additional contribution to the current study. Although these parameters are arrhythmia markers, and we showed that QTc and the frontal QRS-T angle increased after surgery, arrhythmias were not observed in any of the pregnant women in the current study. If our study had a large population, including high-risk pregnant women with heart disease, a comparison between pregnant women with and without arrhythmic events could have been performed.

## 5. Conclusions

In the current study, it was detected that the QTc interval and frontal QRS-T angle were significantly higher in pregnant women with a BMI ≥ 30 than in pregnant women with a BMI < 30. In addition, after a cesarean section with spinal anesthesia, the QT, QTc, and frontal QRS-T angle were significantly increased in the BMI ≥ 30 group, whereas they did not change significantly in the BMI < 30 group. Therefore, it is suggested that close post-operative monitoring is performed in pregnant women with a BMI ≥ 30 undergoing a cesarean section with spinal anesthesia.

## Figures and Tables

**Table 1:** Comparison of basal and clinical variables according to BMI groups.

	BMI < 30 (n = 66)	BMI ≥ 30 (n = 24)	*p*
Age	27.1 ± 4.0	27.4 ± 4.9	0.802 ^a^
Body mass index (kg/m^2^)	27.1 ± 2.0	31.4 ± 1.5	<0.001 ^a^
Gravida	4 (3–5)	4 (4–5)	0.156 ^b^
Duration of operation, min.	27.5 (25–30)	27.5 (25–30)	0.903 ^b^
Gestational week	37 (36–38)	38 (37–38)	0.473 ^b^
Previous number of cesarian	2 (1–2)	2 (1–2)	0.672 ^b^
White blood cell (×10^3^/μL)	10.1 ± 2.4	9.1 ± 2.3	0.075 ^a^
Hemoglobin (g/dL)	11.7 ± 1.3	10.9 ± 1.7	0.705 ^a^

^a^ Student’s *t* test, ^b^ Mann–Whitney U test.

**Table 2:** Mean arterial pressure and heart rate changes during the operation.

	BMI < 30 (n = 66)	BMI ≥ 30 (n = 24)	*p*
Mean arterial pressure, mmHg Pre-operative 1st minute 5th minute 10th minute 20th minute Post-operative	90.6 ± 13.6 86.7 ± 16.2 77.2 ± 14.2 77.2 ± 14.5 78.7 ± 14.8 80.3 ± 14.1	86.8 ± 16.2 84.0± 15.6 74.0 ± 16.9 74.87 ± 15.1 77.0 ± 13.9 77.3 ± 10.5	0.272 ^a^ 0.400 ^a^ 0.375 ^a^ 0.510 ^a^ 0.613 ^a^ 0.379 ^a^
	*p* < 0.001 ^c^	*p* < 0.001 ^c^	
Heart rate, /min Pre-operative 1st minute 5th minute 10th minute 20th minute Post-operative	100.1 ± 6.2 105.8 ± 10.9 105.4 ± 11.5 102.7 ± 10.6 100.3 ± 10.4 92.4 ± 6.3	101.9 ± 8.9 107.5 ± 12.7 105.2 ± 14.6 105.1 ± 14.2 104.4 ± 11.1 93.0 ± 6.4	0.280 ^a^ 0.547 ^a^ 0.938 ^a^ 0.400 ^a^ 0.124 ^a^ 0.737 ^a^
	*p* < 0.001 ^c^	*p* < 0.001 ^c^	

^a^ Student’s *t* test, ^c^ Repeated measures analysis of variance (ANOVA).

**Table 3:** Comparison of QT, QTc, and frontal QRS-T angle between the groups.

	BMI < 30 (n = 66)	BMI ≥ 30 (n = 24)	*p*
QT, ms Pre-operative Post-operative	358.8 ± 23.5 363.1 ± 25.7	355.6 ± 22.1 371.8 ± 32.9	0.583 ^a^ 0.467 ^a^
	*p* = 0.057 ^d^	*p* = 0.008 ^d^	
QTc, ms Pre-operative Post-operative	412.8 ± 16.3 415.9 ± 16.1	420.6 ± 13.3 431.7 ± 18.3	0.037 ^a^ <0.001 ^a^
	*p* = 0.138 ^d^	*p* = 0.010 ^d^	
Frontal QRS-T angle (°) Pre-operative Post-operative	16.0 (7.8–34.3) 21.0 (12.0–34.0)	24.0 (20.0–41.5) 34.5 (19.5–50.0)	0.045 ^b^ 0.007 ^b^
	*p* = 0.056 ^e^	*p* = 0.031 ^e^	

^a^ Student’s *t* test, ^b^ Mann–Whitney U test, ^d^ Paired sample *t* test, ^e^ Wilcoxon test.

## Data Availability

The original contributions presented in this study are included in the article; further inquiries can be directed to the corresponding author.
